# Psychological Distress Among Healthcare Professionals During the Early Stages of the COVID-19 Outbreak in Low Resource Settings: A Cross-Sectional Study in Bangladesh

**DOI:** 10.3389/fpubh.2021.701920

**Published:** 2021-11-11

**Authors:** Md. Riad Hossain, Muhammad Mainuddin Patwary, Rabeya Sultana, Matthew H. E. M. Browning

**Affiliations:** ^1^Institute of Disaster Management, Khulna University of Engineering & Technology, Khulna, Bangladesh; ^2^Environmental Science Discipline, Life Science School, Khulna University, Khulna, Bangladesh; ^3^Environment and Sustainability Research Initiative, Khulna, Bangladesh; ^4^Department of Parks, Recreation and Tourism Management, Clemson University, Clemson, SC, United States

**Keywords:** coronavirus, psychological impacts, mental health, Asia, Global South

## Abstract

The COVID-19 pandemic has been very destructive to and compromised the functioning of all nations' public health systems. In the absence of a vaccine, healthcare workers have been employed to relentlessly fight against COVID-19. The psychological status of healthcare workers during the pandemic in countries with limited resources, notably Bangladesh, remains unclear. The present study aimed to investigate the psychological states of frontline and non-frontline Bangladeshi healthcare workers during the early stages of the COVID-19 outbreak. An online cross-sectional study was conducted from May 5 to 31, 2020 with 203 respondents. Psychological states were measured with a self-reported numerical scale of fear, the Generalized Anxiety Disorder (GAD-7) scale, and the Patient Health Questionnaire (PHQ-9). The prevalence rates of fear, anxiety, and depression were 60.6, 71.9, and 55.2%, respectively. Compared to non-frontline workers, frontline workers reported higher rates of anxiety (79.0 vs. 67.2%) and depression (65.4 vs. 48.4%). Multivariate logistic regression models showed that working in a public institution, being employed for <5 years, and being over-worked were risk factors for developing psychological distress. Our findings emphasize the need for timely psychological interventions to support the mental well-being of healthcare professionals in Bangladesh.

## Introduction

In late December 2019, a new viral outbreak took root in Wuhan city, Hubei province, China. This severe acute respiratory syndrome coronavirus 2 (SARS-COV-2) virus spread rapidly throughout China and spread to other countries soon thereafter (1–3). On February 11th 2020, the World Health Organization (WHO) and the International Committee on Taxonomy of Viruses named the disease resulting from the virus “COVID-19” (Coronavirus Disease 2019, also known as 2019-nCoV) (4). One month later, COVID-19 was declared a global pandemic by the WHO (5). As of April 26th 2021, there have been 147,679,884 confirmed cases of COVID-19 across 215 countries and more than 3 million of confirmed deaths (6).

The COVID-19 pandemic has caused not only high rates of mortality but also severe, negative effects on the mental health of many populations (7), especially healthcare professionals (8). Past and current pandemics have documented numerous, psychological impacts experienced by healthcare workers during these crises (9–11). The severe acute syndrome respiratory (SARS) epidemic in the early 2000's revealed that hospital employees were vulnerable to psychological distress (12), mental disorders (13), and infection by the virus (14). Studies in China during the early stages of the COVID-19 pandemic demonstrated that healthcare workers were susceptible to depression, anxiety, mental distress, stress, somatization, and insomnia (15, 16).

Both frontline and non-frontline healthcare workers were required to work tirelessly in stressful environments with limited resources and therefore experienced negatively psychological impacts from the virus (17). Frontline healthcare workers more frequently interacted with COVID-19 patients (18, 19) despite the virus being more deadly and transmissible than previous epidemics. Consequently, frontline workers may be more susceptible to psychological impacts—including fear, anxiety, and depression—during the viral outbreak than non-frontline workers.

The psychological impacts of the COVID-19 pandemic on healthcare workers are likely to be more acute in developing countries like Bangladesh where healthcare service capacity is poor and population density is high (20, 21). Bangladesh is the 8th most crowded country in the world but has the lowest healthcare provider-to-patient ratio among South Asian countries except for Bhutan and Afghanistan (22). Correspondingly, healthcare workers had insufficient staff support, testing capacity, and quality personal protective equipment (PPE) during the early stages of the pandemic (21, 23, 24). Private hospitals also initially refused to treat COVID-19 patients, which put extra pressure on frontline healthcare workers in Bangladeshi public hospitals (25). Simultaneously, healthcare professionals in Bangladesh faced social stigma, hatred, labeling as virus carriers, and other negative attitudes during the COVID-19 pandemic (26). These attitudes exacerbated healthcare worker's anxiety and depressive symptom levels (27). Additional strain resulted from patients with flu-like symptoms hiding their contact history with infected people (28, 29) and infected patients trying to escape hospitals (30). Healthcare workers were also likely to be fearful of the virus; as of January 17, 2021, the Bangladesh Doctors Federation confirmed that 8,160 healthcare employees had been infected and 130 physicians/surgeons had died from COVID-19 (31). South Asian countries in general have invested little in the mental health services and telemedicine/tele therapy needed by healthcare workers and the general population during the pandemic (32).

It is critical to assess the mental health of healthcare professionals in Bangladesh so that timely psychological interventions can be implemented. Poor mental health of healthcare workers can impede their performance and patient outcomes (33). Several studies have already documented the mental health consequences of the COVID-19 pandemic among Bangladeshi University students (34, 35), children (36), and the general population (37). However, there is limited research on the psychological impacts of COVID-19 on healthcare professionals in Bangladesh. A study by Barua et al. (38) investigated the anxiety, depression, insomnia and fear of frontline doctors. Khatun et al. (39) examined the anxiety and depression rates as well as associated risk factors of 114 physicians. Another study reported suicidal ideation and behavior of healthcare workers (40). Missing from this literature is a systematic assessment of mental health among both frontline and non-frontline healthcare workers in Bangladesh.

Correspondingly, the current study aimed to investigate the psychological distress among frontline and non-frontline healthcare workers during the early stage of the COVID-19 pandemic in Bangladesh. The study also investigated the differences in levels of fear, anxiety and depression and risk factors associated with such psychological problems between frontline and non-frontline healthcare workers in Bangladesh. We hypothesized that both workers experienced fear, anxiety, and depression during the early stages of the pandemic. Further, we hypothesized that frontline healthcare workers showed more psychological distress than non-frontline workers since frontline healthcare workers had more frequently contact with COVID-19 patients.

## Methods

### Study Design and Respondents

We conducted a cross-sectional study using a convenience sample of Bangladeshi healthcare professionals and an online questionnaire. All healthcare professionals working in Bangladesh and registered by the Bangladesh Medical and Dental Council and, Bangladesh Nursing and Midwifery Council were eligible. The survey instrument was distributed through email listservs, closed Facebook groups, and WhatsApp groups between May 5 and May 31, 2020. Informed consent was received from all respondents. The participants were categorized into the following groups: doctors who passed a Bachelor of Medicine and Bachelor of Surgery (MBBS) and practiced medicine, nurses who provided technical assistance to doctors as well as were involved in administrative work at the hospital, dentists who completed a Bachelor of Dental Surgery (BDS) degree and practiced dentistry, and allied health professionals such as physiotherapists, occupational therapists, mental health counselors and physician assistants. A total of 203 healthcare workers participated in the study. The study was approved and supported by the committee for advanced studies and research of Khulna University of Engineering and Technology, Khulna, Bangladesh.

### Measures

The questionnaire asked respondents about their sociodemographic characteristics, workplace exposure, and three aspects of their mental health. Sociodemographic characteristics included age, gender, and highest level of educational achievement. Residency characteristics included place of residence (urban vs. rural) and co-living status (i.e., with or without family members).

#### Employment Status

Workplace exposure was used to differentiate respondents into frontline and non-frontline workers. Frontline workers were medical staff directly involved with COVID-19 patient care, and non-frontline workers were medical staff without direct involvement with COVID-19 patient care (41). We distinguished these two groups of workers by asking respondents whether they engaged directly with treating COVID-19 patients.

Additional data on healthcare facility type, working institute, work experience, and over-worked status were collected to understand the working conditions of respondents.

#### Psychological Distress

A single item was used to measure self-reported fear levels. Respondents indicated how fearful they were during the COVID-19 pandemic on a scale from 0 (no fear at all) to 10 (extremely fearful). We used a cutoff score of >6 to indicate high levels of fear (42).

The Patient Health Questionnaire (PHQ-9) was used to measure respondent's depression levels over the past 2 weeks. This is a well-validated tool to screen the severity of depressive symptoms and clinical levels of depression (Cronbach's α = 0.89) (43). The PHQ-9 includes nine items that were answered on a 0 (not at all) to 3 (almost every day) response scale. Items were summed to obtain a summary score between 0 and 27. Scores of 0–4 indicated minimal to no depression, 5–9 indicated mild depression, 10–14 indicated moderate depression, and scores of 15–21 indicated severe depression (44). We used these four levels of depression as well as a cutoff score of 10 points or more to identify clinical levels of major depressive disorder (45).

We used the Generalized Anxiety Disorder 7-item (GAD-7) scale to assess anxiety levels (46). This is a commonly-used screening tool with excellent validity and reliability (Cronbach's α = 0.911) (46). Respondents indicated the frequency of anxiety symptoms over the past 2 weeks on a 0 (not at all) to 3 (almost every day) response scale. A summary score was created by summing all items. Respondents were categorized as having minimal/no anxiety (summary scores between 0 and 4), mild anxiety (5–9), moderate anxiety (10–14), or severe anxiety (15–21) (46). In addition to these four levels of anxiety, we used a cutoff score of nine points or more to identify clinical levels of generalized anxiety disorder (47).

### Analysis

Descriptive statistics were used to analyze the demographic characteristics of respondents. Categorical variables were presented as percentages and continuous variables were presented as means (±standard deviations). We checked for normality of the mental health outcomes using the Shapiro-Wilk test (48). The data did not meet normality (*p* < 0.05) so non-parametric tests were used for subsequent analyses. Variables were compared between frontline and non-frontline healthcare workers by using χ^2^ and Kruskal-Wallis tests. Univariate and multivariable logistic regression models identified potential predictors of psychological distress. Statistically significant predictors in the univariate analysis were used for the multivariate logistic regression models. Models were adjusted for age, gender, highest level of educational achievement, current place of residence, living status, healthcare type, type of workplace, years of employment and daily working hours. A *p*-value of <0.05 was considered to be statistically significant. Analyses were conducted in the R statistical software package (version 4.0.0) and SPSS statistical software (version 21).

## Results

### Sample Characteristics

Table 1 summarizes the descriptive statistics of the respondents. A total of 203 healthcare workers participated including 150 doctors, 24 nurses, 22 dentists, and seven allied health professionals. Of these, ~50% (*N* = 97) were women. The mean (sd) age of respondents was 33.12 (±9.14) and the vast majority (>95%) lived in an urban area. Approximately 85% (*N* = 172) of respondents resided with their families during the pandemic. Most respondents (52.2%) had attained a graduate level of education; fewer numbers of respondents had attained only postgraduate studies (33%), undergraduate degrees (7.39%), advanced degrees (4.43%), or an uncompleted college degree (2.96%). Approximately 75% of respondents (*N* = 121) worked for public hospitals and 52.22% (*N* = 106) had worked for <5 years after their terminal degree. More than 83% (*N* = 169) of respondents worked 8 h or more per day. Approximately 40% (*N* = 81) were frontline workers during the COVID-19 pandemic.

**Table 1:** Descriptive statistics of respondents' socio-demographic characteristics, residency, and employment status.

**Variables**	**Total**	**Frontline healthcare workers**	**Non-frontline healthcare workers**	**χ^2^^a^**	***P*-value**
	**(*N* = 203)**	**(*N* = 81)**	**(*N* = 122)**		
**Gender**				0.41	0.84
Male	106 (52.22)	43 (53.09)	63 (51.64)		
Female	97 (47.78)	38 (46.91)	59 (48.36)		
**Age**	33.12 (±9.14)	34.12 (±9.55)	32.45 (±8.84)	0.75	0.38
**Place of residence**				0.08	0.78
Urban	194 (95.57)	77 (95.06)	117 (95.90)		
Rural	9 (4.43)	4 (4.94)	5 (4.10)		
**Living Status**				4.13	0.12
With family members	172 (84.73)	73 (90.12)	99 (81.15)		
With non-family members	24 (11.82)	5 (6.17)	19 (15.57)		
Alone	7 (3.45)	3 (3.70)	4 (3.28)		
**Education**				7.86	0.039
College	6 (2.96)	1 (1.23)	5 (4.10)		
Undergraduate	15 (7.39)	3 (3.70)	12 (9.84)		
Graduate	106 (52.22)	40 (49.38)	66 (54.10)		
Postgraduate	67 (33)	31 (38.27)	36 (29.51)		
Advanced degree (MPhil, Ph.D.)	9 (4.43)	6 (7.41)	3 (2.46)		
**Healthcare sector**				11.79	0.008
Doctor	150 (73.89)	70 (86.42)	80 (65.57)		
Nurse	24 (11.82)	6 (7.41)	18 (14.75)		
Dentist	22 (10.84)	3 (3.70)	19 (15.57)		
Allied health	7 (3.45)	2 (2.47)	5 (4.10)		
**Type of healthcare workplace**				1.19	0.28
Public	121 (59.61)	52 (64.20)	69 (56.56)		
Private	82 (40.39)	29 (35.80)	53 (43.44)		
**Years of employment**				4.21	0.12
<5 years	106 (52.22)	36 (44.44)	70 (57.38)		
5–9 years	46 (22.66)	19 (23.46)	27 (22.13)		
>9 years	51 (25.12)	26 (32.10)	25 (20.49)		
**Working hours**				0.86	0.65
<8 h/day	34 (16.75)	13 (16.05)	21 (17.21)		
≥8 h/day	169 (83.25)	68 (83.95)	101 (82.79)		

*Data are presented as N (%) or mean (±SD)*.

a *Kruskal-Wallis test*.

Frontline and non-frontline healthcare workers showed different levels of education (χ^2^ = 7.86, df = 3, *p* < 0.05) and healthcare sector (χ^2^ = 11.79, df = 3, *p* < 0.05). Frontline healthcare workers had achieved higher levels of education including postgraduate and advanced degrees than non-frontline healthcare workers (45.67 vs. 31.96%). A larger share of physicians was present in the non-frontline healthcare worker group than in the frontline healthcare group (86.4 vs. 65.6%). No other significant differences were observed in respondent's socio-demographic characteristics, residency, or employment status (Table 1).

### Psychological Distress Levels Among Healthcare Workers

Table 2 illustrates the psychological states of healthcare workers. Prevalence rates across the sample of respondents were 60.59, 71.92, and 55.17% for fear, anxiety, and depression, respectively. Prevalence rates were different between frontline and non-frontline workers for anxiety (χ^2^ = 4.16, df = 1, *p* < 0.05) and depression (χ^2^ = 4.89, df = 1, *p* < 0.05) but for not fear (χ^2^ = 0.29, df = 1, *p* >0.05). More frontline than non-frontline workers reported having anxiety (79.01 vs. 67.21%) and depression (65.43 vs. 48.36%). Greater shares of frontline workers had high anxiety levels than non-frontline workers (27.16 vs. 13.93%). Similarly, more frontline workers had high depression levels than non-frontline workers (29.63 vs. 15.57%). We observed no significant difference in fear levels between frontline and non-frontline workers (*p* > 0.05).

**Table 2:** Psychological states of Bangladeshi healthcare workers during the early stages of the COVID-19 pandemic (*N* = 203).

**Variables**	**Total**	**Frontline healthcare workers**	**Non-frontline healthcare workers**	**χ^2^^a^**	***P*-value**
	**(*N* =203)**	**(*N* = 81)**	**(*N* = 122)**		
**Fear**				0.29	0.58
>6	123 (60.59)	50 (61.72)	73 (59.83)		
**Anxiety (GAD-7)**				4.16	0.04
Minimal	17 (8.37)	4 (4.94)	13 (10.66)		
Mild	52 (25.62)	22 (27.16)	30 (24.59)		
Moderate	95 (46.80)	33 (40.74)	62 (50.82)		
Severe	39 (19.21)	22 (27.16)	17 (13.93)		
Clinical level of Generalized Anxiety Disorder (≥9)	146 (71.92)	64 (79.01)	82 (67.21)		
**Depression (PHQ-9)**				4.89	0.02
Minimal	30 (14.78)	11 (13.58)	19 (15.57)		
Mild	61 (30.05)	17 (20.99)	44 (36.07)		
Moderate	69 (33.99)	29 (35.80)	40 (32.79)		
Severe	43 (21.18)	24 (29.63)	19 (15.57)		
Clinical level of Major Depressive Disorder (≥10)	112 (55.17)	53 (65.43)	59 (48.36)		

*Data presented as N (%)*.

a *Kruskal-Wallis test*.

### Risk Factors for Psychological Distress Among Healthcare Workers

Table 3 presents the univariate analyses of risk factors associated with psychological distress. Living status, education, type of healthcare workplace, years of employment, and working hours were significantly associated with the psychological distress. Specifically, respondents living with non-family members (*F* = 3.45, *p* < 0.05) or working in a public institute (*F* = 5.63, *p* < 0.05) had higher levels of fear than others. In terms of the GAD-7, healthcare workers who received college levels of education (*F* = 2.78, *p* < 0.05), worked in a public healthcare facility (*F* = 4.51, *p* < 0.05), completed 5–9 years of emp*l*oyment (*F* = 5.48, *p* < 0.001) or worked ≥8 h per day (*F* = 8.71, *p* < 0.001) had higher scores of anxiety than others. Regarding the PHQ-9, the respondents who worked in a public healthcare facility (*F* = 2.16, *p* < 0.05) or worked ≥8 h per day (*F* = 3.56, *p* < 0.05) had higher levels of depression than others.

**Table 3:** Univariate analysis of risk factors associated with psychological disorder among Bangladeshi healthcare workers during the early stages of the COVID-19 pandemic (*N* = 203).

**Variables**	**Fear**		**Anxiety**		**Depression**	
	***N* (%)**	***F***	***N* (%)**	***F***	***N* (%)**	***F***
**Gender**						
Male	61 (57.55)	1.68	75 (70.75)	0.15	62 (58.49)	0.05
Female	62 (63.92)		71 (73.20)		50 (51.55)	
**Age**	34.98 ± 10.14	1.19	34.29 ± 9.50	1.23	33.82 ± 9.30	1.01
**Place of residence**						
Urban	116 (59.79)	0.09	140 (72.16)	0.76	108 (55.67)	1.75
Rural	7 (77.78)		6 (66.67)		4 (44.44)	
**Living Status**						
With family members	103 (59.88)	3.45^*^	123 (71.51)	0.53	93 (54.07)	0.09
With non-family members	18 (75.00)		18 (75.00)		14 (58.33)	
Alone	2 (28.57)		5 (71.43)		5 (71.43)	
**Education**						
College	3 (50.00)	0.42	5 (83.33)	2.78^*^	3 (50.00)	1.32
Undergraduate	9 (60.00)		12 (80.00)		9 (60.00)	
Graduate	59 (55.66)		71 (66.98)		54 (50.94)	
Postgraduate	44 (65.67)		52 (77.61)		42 (62.69)	
Advanced degree (MPhil, Ph.D.)	8 (88.89)		6 (66.67)		4 (44.44)	
**Healthcare sector**						
Doctor	91 (60.67)	1.14	104 (69.33)	0.46	81 (54.00)	0.13
Nurse	17 (70.83)		19 (79.17)		14 (58.33)	
Dentist	14 (63.64)		16 (72.73)		12 (54.55)	
Allied health	1 (14.29)		7 (100)		5 (71.43)	
**Type of healthcare workplace**						
Public	80 (66.12)	5.63^*^	92 (76.03)	4.51^*^	70 (57.85)	2.16^*^
Private	43 (52.44)		54 (65.85)		42 (51.22)	
**Years of employment**						
<5 years	57 (53.77)	0.48	68 (64.15)	5.48^***^	54 (50.94)	0.51
5–9 years	23 (50.00)		36 (78.26)		28 (60.87)	
>9 years	43 (84.31)		42 (82.35)		30 (58.82)	
**Working hours**						
<8 h/day	19 (55.88)	0.72	17 (50.00)	8.71^***^	18 (52.94)	3.56^*^
≥8 h/day	104 (61.54)		129 (76.33)		94 (55.62)	

*Cutoffs included >6 for fear, ≥9 for anxiety on the GAD-7, and ≥10 on the PHQ-9 for depression*.

* *p < 0.05*,

*** *p < 0.001*.

Table 4 shows the logistic regression models results, which identified risk factors of psychological distress when all significant variables in the univariate analyses were considered simultaneously. Notably, frontline workers who reported working ≥8 h/day were 2.5 times as likely to report high levels of anxiety [OR = 2.5, 95% CI (0.52–12.33), *p* < 0.05] and 36% more likely to report high levels of depression [OR = 1.36, 95% CI (0.39–5.20), *p* < 0.05]. Frontline workers who lived with non-family members were 88% less likely to report high levels of fear than frontline workers living alone [OR = 0.02, 95% CI (0.00–1.13), *p* < 0.05]. Frontline workers who worked for <5 years were 30% more likely to have high levels of anxiety [OR = 1.30, 95% CI (0.24–6.26), *p* < 0.05].

**Table 4:** Regressing socio-demographic and work conditions on high levels of psychological distress (fear, anxiety, and depression) among Bangladeshi healthcare frontline and non-frontline workers.

**Variables**	**Frontline healthcare workers (*****N*** **=** **81)**			**Non-frontline healthcare workers (*****N*** **=** **122)**		
	**Model 1 (Fear)**	**Model 2 (Anxiety)**	**Model 3 (Depression)**	**Model 4 (Fear)**	**Model 5 (Anxiety)**	**Model 6 (Depression)**
	**OR (95% CI)**	**OR (95% CI)**	**OR (95% CI)**	**OR (95% CI)**	**OR (95% CI)**	**OR (95% CI)**
**Living status**						
With family members	0.01 (0.00–2.86)	-	-	0.37 (0.02–3.35)	-	-
With non-family members	0.02 (0.00–1.13)^*^	-	-	0.36 (0.01–4.06)^*^	-	-
Alone	Ref.	-	-	Ref.	-	-
**Education**						
College	-	7.86 (0.36–391.4)	-	-	10.35 (0.28–372.03)	-
Undergraduate	-	4.52 (0.95–549.71)	-	-	0.73 (0.03–16.08)	-
Graduate	-	4.40 (0.35–108.4)	-	-	0.95 (0.05–15.08)	-
Postgraduate	-	4.53 (0.37–108.7)	-	-	1.8 (0.10–30.90)	-
Advanced degree	-	Ref.	-	-	Ref.	-
**Type of healthcare workplace**						
Public	0.70 (0.22–2.19)	1.30 (0.56–2.99)	1.01 (0.32–3.17)	3.70 (1.63–8.64)^***^	1.30 (0.56–2.97)	1.15 (0.54–2.46)
Private	Ref.	Ref.	Ref.	Ref.	Ref.	Ref.
**Years of employment**						
<5 years	-	1.34 (0.24–6.26)^*^	-	-	1.44 (0.44–4.63)	-
5–9 years	-	1.01 (0.16–3.32)	-	-	0.66 (0.16–2.66)	-
>9 years	-	Ref.	-	-	Ref.	-
**Working hours**						
<8 h	-	Ref.	Ref.	-	Ref.	Ref.
≥8 h	-	2.5 (0.52–12.33)^*^	1.36 (0.39–5.20)^*^	-	0.01 (0.00–0.21)	0.00 (0.00–0.01)

*Results of logistic regression with cutoffs of >6 for fear, ≥9 for anxiety on the GAD-7, and ≥10 on the PHQ-9 for depression*.

* *p < 0.05;*

*** *p < 0.001*.

*OR, odds ratio; CI, 95% confidence interval*.

*Bold values represented as significant variables*.

In contrast, frontline workers who worked ≥8 h/day were 81% *less* likely to have high levels of anxiety [OR = 0.09, 95% CI (0.00–0.11), *p* < 0.001]. Working hours did not influence the odds of having any psychological distress (*p* > 0.05) among non-frontline workers. Non-frontline workers were 64% less likely to report high levels of fear if they lived with non-family members vs. living alone [OR = 0.36, 95% CI (0.01–4.06), *p* < 0.05]. Also, non-frontline workers who were employed in public healthcare facilities were 3.7 times more likely to report high levels of fear than those employed in private facilities [OR = 3.70, 95% CI (1.63–8.64), *p* < 0.001].

## Discussion

### Summary and Interpretation of Main Findings

The COVID-19 pandemic has brought about a global public health crisis and healthcare professionals have been playing a frontline role in combating the pandemic (49). Although several psychological consequences of the COVID-19 pandemic among healthcare workers have been speculated on, the relevance of assessing the psychological burden and COVID-19-related issues of healthcare professionals in developing countries like Bangladesh remains of great importance (50). In comparison to earlier epidemics such as SARS, the psychological state of medical professionals during COVID-19 is particularly concerning (18, 51). Bangladesh is no exception to this and its healthcare workers have experienced elevated levels of fear, depression, and anxiety due to high infection rates, the lack of sufficient medical personnel, the shortage of healthcare resources, and the inadequate supply of quality protective equipment among other societal problems (52). The current study is the first to directly compare psychological distress among both frontline and non-frontline healthcare workers during the early stages of the COVID-19 outbreak in Bangladesh.

Our study confirms that both frontline and non-frontline healthcare workers encountered severe psychological distress during the early outbreak of the disease in Bangladesh. However, the prevalence rates of fear, anxiety, and depression were noticeably higher among frontline healthcare workers than among non-frontline workers. A concerningly-high share of frontline healthcare workers reported clinical levels of anxiety (79.0%) and depression (67.2%). These results are reinforced by other recent studies among frontline workers in other countries (41, 53, 54). Specifically, Alshekaili et al. (53) found that the prevalence of anxiety, stress, and insomnia was 1.5 times higher in frontline healthcare workers than in non-frontline employees in Oman. Cai et al. (54) reported that 52.6% of frontline workers experienced psychological distress whereas only 34.0% of non-frontline workers reported psychological distress in China. Another study also found high levels of anxiety and depressive symptoms amongst frontline medical workers in China (41). Like our study, Tan et al. (55) found even non-frontline workers in Singapore where infection rates were low reported high levels of anxiety although no comparison to frontline workers was made (45). Our findings also align with the psychological impacts on healthcare workers during previous epidemics (56–58).

The high prevalence rates of anxiety and depression among frontline healthcare workers can be explained by myriad factors. Notably, the hospitals in Bangladesh and elsewhere were overcrowded environments that impacted mental health (59). Healthcare workers also had inadequate personal protective equipment (PPE) supplies (60) of questionable quality (61). Further, patients with potential COVID-19 symptoms sometimes fled from the hospital (28, 29), which could have further impacted frontline workers. Similarly, some patients with flu-like symptoms tended to hide their travel and contact history, making it difficult to treat patients smoothly (30). Social problems may have played a role in the psychological states of frontline workers as well. In the absence of an effective vaccine protocol, people often succumbed to invalidated homeopathic therapies to cure COVID-19 such as consuming Asian pennywort leaves (locally called Thankuni), drinking tea or warm water with ginger or garlic (62). Such misinformation could have put additional strain on the mental health of healthcare workers.

We found that non-frontline healthcare workers who worked in a public healthcare facility were more likely to have high levels of fear than non-frontline workers working in a private facility. Initially, only public hospitals permitted COVID-19 treatments in Bangladesh (63). The majority of these hospitals had shortages in PPE, ICU beds, ventilation units, and medical personnel (64), and patients with flu-like fevers often hid their contact history (30). These situations made it very difficult, if not impossible, to treat normal patients. In addition, the private hospitals refused to treat suspected COVID-19 patients, and readily referred such patients to government hospitals (64). Such a large number of patients made an already overcrowded environment in the emergency room impossible to maintain social distancing or posed serious challenges to practicing personal safety (tear-off workers' mask) for healthcare workers (65). Consequently, healthcare workers became fearful of contagions with the coronavirus. Consequently, the findings of this study should persuade the government to ensure adequate protective equipment for healthcare workers is provided promptly, in order to reduce the fear of getting infected and in turn save their general mental health well-being.

We observed that over-worked status was strongly associated with the psychological distress of the frontline. The frontline healthcare workers who worked at least 8 h per day were much likely to experience anxiety and depression than those who worked fewer hours per day. This finding is consistent with the growing evidence that demonstrates how long working hours is associated with poor mental health (16, 41, 66). For example, Moazzami et al. (67) reported that frontline healthcare workers faced unprecedented workloads during the COVID-19 pandemic and this overworked status may have led to emotional exhaustion. Depression, anxiety, and stress have also been associated with increased weekly working hours during COVID-19 (68), and a recent study in Iran reported that frontline nurses with higher workloads during COVID-19 experienced worse mental health than other healthcare staff (69). These findings collectively support policies regarding reasonable numbers of working hours and giving healthcare staff sufficient rest periods and/or shift work to prevent severe mental health issues and burnout.

We also found that living with non-family members decreased the odds of high fear levels among frontline and non-frontline workers compared with living alone. Numerous studies have previously found that the lack of contact/communication with family members or friends was associated with the development of psychological problems (45, 70, 71). We did not find that living with family members reduced the odds of high fear levels, suggesting that respondents may have been concerned about possible asymptomatic infection from family but not other co-habitants, such as fellow healthcare workers (72).

Our research found that years of employment were associated with the anxiety levels of frontline workers. New and younger workers are more likely to develop psychiatric problems during public health emergencies (73). One of the possible reasons for this finding may be due to simply not having previously experienced a public health emergency. Consequently, the younger workers were prone to remaining isolated in their room and to prevent physical interactions in fear of contagion with the virus, especially in high-risk working environments, contributing to psychological trauma (74). A recent study by Elbay et al. (68) reported that the younger frontline healthcare workers who had worked for less time produced a high score of depression, anxiety, and stress. In contrast, more experienced staff who had worked in prior epidemics such as SARS, H1N1, and MERS were already alerted to the need for self-protection, cleanliness, quarantine, etc., so they exhibited better confidence and mental well-being compared to younger healthcare professionals (75). Therefore, regular consultation with peers either in real or virtual platform, online mental health counseling, regular mental health assessment, ensuring the availability of adequate mental health resources, and access to professional mental health training for young staff could ameliorate the mental health of the healthcare workers during a pandemic (70, 76).

### Strengths and Limitations

The primary strength of this study is its novel investigation of both frontline and non-frontline healthcare workers during the early stages in the COVID-19 pandemic in a resource-limited country with an extremely dense population. The limitations of this study include the modest sample size, which may not have been representative of all healthcare workers in Bangladesh. Further, the sample may have been biased toward certain respondents who could access the internet. This research was conducted during the early stages of the COVID-19 outbreak in Bangladesh and lacked longitudinal follow-up data. We were unable to predict psychological distress rates in other stages of the pandemic. Although this research controlled for important socio-demographic, residency, and employment characteristics, there may be additional confounding factors such as social support, comorbidities, family history of mental illness, and life events. Finally, the cross-sectional nature of our study prevented drawing any cause-and-effect relationships between risk factors and mental health. Despite having these limitations, this study from a nationwide sample provided useful insights regarding the early psychological impacts of COVID-19 on healthcare workers by using globally validated mental health tools.

### Policy Recommendation

Although the Bangladesh government provided various stimulus packages for healthcare workers, the proper allocation of mental health services should continue to be given the highest priority. Since the Bangladesh already witnessed a huge toll of cases in the second wave of COVID-19, the findings of this study could help the government design appropriate strategies to reduce the psychological burdens on healthcare workers. Specifically, the government could consider establishing a multidisciplinary team for mental health surveillance with qualified and specialized mental health practitioners so that healthcare workers could communicate their psychological concerns. Furthermore, the hospitals could encourage shiftwork so that frontline and non-frontline staff have enough rest and time to recuperate. An observation of previous epidemics and pandemics highlights that COVID-19 disrupted the mental health of healthcare workers once the pandemic struck and showed no signs of abating (77, 78). Psychosocial interventions should be introduced to the individuals who suffer from the consequences of COVID-19 to improve their mental well-being during the post-pandemic period. In the least, routine mental health screening should be made available by professional psychiatrists to healthcare workers.

## Conclusion

In conclusion, the COVID-19 pandemic has caused a heavy psychological impact on frontline healthcare workers. In our study, frontline healthcare workers showed higher levels of anxiety and depression compared to non-frontline healthcare workers during the COVID-19 pandemic in Bangladesh. Additionally, our findings showed that lack of work experience and excessive workloads were associated with negative mental health outcomes for frontline healthcare workers. Thus, a timely psychological interventions along with virus knowledge development programs should be implemented immediately to reduce the mental disorder of and improve the mental well-being of healthcare workers in Bangladesh.

## Data Availability Statement

The raw data supporting the conclusions of this article will be made available by the authors, without undue reservation.

## Ethics Statement

The study followed the process of the Declaration of Helsinki and maintained the highest possible extent of ethical standards. An electronic consent of participation was obtained from all the respondents before they took part in the study. The study was approved by the Ethical Review Committee for Advanced Studies and Research, Khulna University of Engineering and Technology, Khulna-9203, Bangladesh.

## Author Contributions

MH: conceptualization, data curation, investigation, and writing—review and editing. MP: conceptualization, methodology, formal analysis, and writing—original draft. RS: conceptualization, data curation, and writing—review and editing. MB: writing—review and editing. All authors contributed to the article and approved the submitted version.

## Funding

This research has been supported by the Committee for Advanced Studies and Research of Khulna University of Engineering and Technology, Khulna, Bangladesh.

## Conflict of Interest

The authors declare that the research was conducted in the absence of any commercial or financial relationships that could be construed as a potential conflict of interest.

## Publisher's Note

All claims expressed in this article are solely those of the authors and do not necessarily represent those of their affiliated organizations, or those of the publisher, the editors and the reviewers. Any product that may be evaluated in this article, or claim that may be made by its manufacturer, is not guaranteed or endorsed by the publisher.
